# Transcriptome Signatures Predict Phenotypic Variations of *Candida auris*

**DOI:** 10.3389/fcimb.2021.662563

**Published:** 2021-04-14

**Authors:** Sabrina Jenull, Michael Tscherner, Nataliya Kashko, Raju Shivarathri, Anton Stoiber, Manju Chauhan, Andriy Petryshyn, Neeraj Chauhan, Karl Kuchler

**Affiliations:** ^1^ Max Perutz Labs Vienna, Department of Medical Biochemistry, Medical University of Vienna, Vienna, Austria; ^2^ Public Health Research Institute & Department of Microbiology, Biochemistry and Molecular Genetics, New Jersey Medical School, Rutgers, The State University of New Jersey, Newark, NJ, United States

**Keywords:** *Candida auris*, transcriptional profiling, phenotypic variation, antifungal multidrug resistance, RNA-seq

## Abstract

Health care facilities are facing serious threats by the recently emerging human fungal pathogen *Candida auris* owing to its pronounced antifungal multidrug resistance and poor diagnostic tools. Distinct *C. auris* clades evolved seemingly simultaneously at independent geographical locations and display both genetic and phenotypic diversity. Although comparative genomics and phenotypic profiling studies are increasing, we still lack mechanistic knowledge about the *C. auris* species diversification and clinical heterogeneity. Since gene expression variability impacts phenotypic plasticity, we aimed to characterize transcriptomic signatures of *C. auris* patient isolates with distinct antifungal susceptibility profiles in this study. First, we employed an antifungal susceptibility screening of clinical *C. auris* isolates to identify divergent intra-clade responses to antifungal treatments. Interestingly, comparative transcriptional profiling reveals large gene expression differences between clade I isolates and one clade II strain, irrespective of their antifungal susceptibilities. However, comparisons at the clade levels demonstrate that minor changes in gene expression suffice to drive divergent drug responses. Finally, we functionally validate transcriptional signatures reflecting phenotypic divergence of clinical isolates. Thus, our results suggest that large-scale transcriptional profiling allows for predicting phenotypic diversities of patient isolates, which may help choosing suitable antifungal therapies of multidrug-resistant *C. auris.*

## Introduction

Infectious diseases pose a major threat to human health, which is further driven by the ever-ongoing emergence of new pathogens (Satoh et al., 2009; Cloeckaert and Kuchler, 2020; Wu et al., 2020). The increasing prevalence of (multi)drug-resistant microbial pathogens (Blair et al., 2015; Berman and Krysan, 2020) constitutes a worrisome global trend, which requires immediate attention in basic and applied research. SARS-Cov-2 is a horrific example of what can happen when treatment of a pathogen with pandemic potential is unavailable or when health care is forced into the “reactive” mode rather than the essential “preparedness” mode that would ensure eradication of pandemics (Cloeckaert and Kuchler, 2020). Ever since the first reported case of the human fungal pathogen *Candida auris* (Satoh et al., 2009), it has caused hospital outbreaks in more than 40 countries by today (Du et al., 2020). The rapid and independent emergence of *C. auris* in different geographical locations (Lockhart et al., 2017; Chow et al., 2020), along with its pronounced pan-antifungal traits has sparked serious concerns. Based on genomic analysis and the initial occurrence, *C. auris* clusters into 4 major clades, with the most recent emergence of a possible fifth clade from Iran (Chow et al., 2019). Isolates from different clades are genetically diverse, though intra-clade variability is limited at the genomic level, suggesting a clonal expansion for each clade (Sharma et al., 2016; Muñoz et al., 2018). In addition to global spreading of *C. auris*, this pathogen constitutes an epic clinical challenge due to poor diagnostics and because of its dramatic antifungal multidrug resistance (MDR) (Chowdhary et al., 2017; Mizusawa et al., 2017; Kordalewska and Perlin, 2019).

To date, four main classes of antifungals are in clinical use: azoles, echinocandins, polyenes and nucleoside analogues (Berman and Krysan, 2020). While the majority of identified *C. auris* isolates display intrinsic resistance to azoles such as fluconazole, susceptibilities to other antifungals strongly vary among clades. Recent studies demonstrated that clade II isolates (East Asian clade) show the highest degree of antifungal susceptibilities, while clade I strains (South Asian clade) show the lowest susceptibility (Arendrup et al., 2017; Szekely et al., 2019; Chen et al., 2020; Chow et al., 2020). In line with this, resistance to the polyene amphotericin B (AmB) was mainly detected in clade I and clade IV (South America clade) isolates (Chow et al., 2020). Generally, about 15-30% of *C. auris* isolates exhibit a high tolerance to AmB and 2-8% of isolates are echinocandin-resistant (Chowdhary et al., 2017), making echinocandins the choice of first-line treatment. However, isolates resistant to more than 2 or all classes of antifungals and isolates from India that show a severe increase in echinocandin-resistant have been observed (Kathuria et al., 2015; Chowdhary et al., 2017; Kordalewska et al., 2018; Chen et al., 2020).

In addition to pronounced MDR traits, *C. auris* shows strong adhesion to biotic surfaces such as human skin (Schelenz et al., 2016; Chow et al., 2018; Horton et al., 2020), which has become the major route of person-to-person transmission in health care settings (Forsberg et al., 2019). Therefore, the Centers of Disease Control and Prevention (CDC) has recently recognized the pandemic potential, and classified it as a global threat to human health, thus requiring urgent attention in medicare for the next decade (https://www.cdc.gov/fungal/candida-auris; (Meis and Chowdhary, 2018). Recent studies provide first glimpses about evolution, morphogenetic plasticity, host interactions and skin colonization behavior of different *C. auris* clades (Muñoz et al., 2018; Yue et al., 2018; Bruno et al., 2020; Chow et al., 2020; Huang et al., 2020). However, basic knowledge about *C. auris* cell biology or molecular mechanisms of its phenotypic diversification are lagging behind.

Cell fate and adaptations to environmental cues are not only determined by genomic variations, but are also fundamentally affected by regulators of epigenetic and transcriptional signatures (Burton and Torres-Padilla, 2014; Lappalainen and Greally, 2017). Likewise, alterations in these programs drive divergent phenotypic diversification that allows for niche adaptations, even under immune surveillance in host settings (Gómez-Díaz et al., 2012; Huang et al., 2019). Therefore, we aimed to assess transcriptomic profiles of different clade I isolates and the Japanese clade II type strain (CBS10913 aka CDC B11220) to identify inter- but also intra-clade-specific gene expression patterns linked to distinct phenotypical properties. In this study, we present transcriptional signatures of *C. auris* clade I isolates with distinct antifungal susceptibility profiles. We demonstrate that a limited set of regulated genes can drive divergent antifungal responses. In addition, we provide experimental validation of top-regulated genes mediating azole susceptibility and proteolysis-mediated growth of *C. auris* isolates. Hence, we believe that comparative transcriptional profiling of a variety of *C. auris* isolates will aid to predict fungal biological properties as well as MDR phenotypes based on gene expression signatures.

## Material and Methods

### Media and Fungal Growth Conditions

All strains used in this study are listed in **Supplementary Table 1**. Clinical isolates of *C. auris* were a generous gift of Rajendra Prasad and Arunoloke Chakraborti. Candida strains were routinely grown on YPD medium (1% yeast extract, 2% peptone and 2% glucose [all BD Biosciences]) at 30°C with 200 rpm shaking. For solid medium, 2% Bacto agar (BD Biosciences) was added. Synthetic complete (SC; 1.7 g/L yeast nitrogen base without amino acids and ammonium sulfate [BD Biosciences], 5 g/L ammonium sulfate [Sigma-Aldrich], amino acid mix and 2% glucose [all BD Biosciences]) medium was prepared as previously described (Kaiser et al., 1994). YCB-BSA medium was composed of 23.4 g/L yeast carbon base (Sigma-Aldrich) pH-adjusted with HCl to 4.0 and 5 g/L BSA (Sigma-Aldrich).

### Antifungal Susceptibility Screening on Solid Agar Medium

For antifungal susceptibility testing on solid medium, fungal strains were printed on solid YPD medium from cryo-cultures arranged on a 96-well plate using a robot instrument (RoToR HDA, Singer Ltd., Roadwater, UK). YPD plates were then incubated at 30°C for 3 days and material form fungal colony spots were inoculated in 200 µl liquid YPD medium in a 96-well plate using the robot instrument. Cultures were grown overnight at 30°C with constant agitation (150 rpm) and spotted on solid SC medium with or without antifungal drugs using the robot instrument. Plates were imaged after 3 days at 30°C. Plate spotting was performed in duplicates. The colony size was calculated using the R “gitter” package (https://github.com/omarwagih/gitter) and the relative colony size represents the colony size ratio on SC supplemented with antifungals relative to the colony size on SC medium alone. The lower the ratio of colony size with drug vs no drug, the more susceptible is the isolate. The following antifungals were used: fluconazole (FCZ; Discovery Fine Chemicals Ltd; 64, 32, 16, 8 and 4 µg/ml in DMSO [Sigma-Aldrich]), itraconazole (ICZ; Discovery Fine Chemicals Ltd; 0.15, 0.075 µg/ml in DMSO), voriconazole (VCZ; Discovery Fine Chemicals Ltd; 0.15, 0.075 µg/ml in DMSO), amphotericin B (AmB; Santa Cruz Biotechnology; 3.0 and 1.5 µg/ml in DMSO), caspofungin (Casp; Merck; 0.40, 0.20, 0.12 µg/ml in dH_2_O), 5-fluorocytosine (5-FC; Sigma-Aldrich; 10 and 5 µg/ml in dH_2_O).

### Growth Inhibition Assays

Antifungal susceptibility of selected *C. auris* isolates was confirmed using a minimal inhibitory concentration (MIC) assay in liquid YPD medium as described previously (Schwarzmüller et al., 2014) with minor modifications. Briefly, fungal cells were grown overnight in YPD at 30°C with constant agitation (200 rpm) and regrown the next day in fresh YPD medium at 30°C, 200 rpm to an OD_600_ of approximately 1. Cells were then further diluted in YPD medium to an inoculum of 2.5 x 10^4^ cells per ml. Antifungal stocks were serially diluted 1:2 in YPD and 100 µl of each dilution were suspended into a well of a 96-well plate. Control wells with YPD medium only or supplemented with the corresponding DMSO (Sigma-Aldrich) concentration (2% final) were included, if the tested antifungal was prepared in DMSO. Hundred µl of inoculum were then added and plates were incubated at 30°C for 24 hours followed by OD_600_ readings using a Victor Nivo plate reader (PerkinElmer). Wells without cells served as blank control. Values for the 50% inhibitory concentration (IC_50_) were calculated with a 4-parameter log-logistic model using the R “drc” package (Ritz et al., 2016).

### Generation of *CDR1* Gene Deletion Mutants

The *C. auris* homologue of *C. albicans CDR1* was deleted using a fusion PCR strategy exactly as published previously (Schwarzmüller et al., 2014). Briefly, roughly 500 bp flanking regions upstream and downstream of *C. auris CDR1* were amplified from genomic DNA (gDNA) extracted from the CBS10913 strain as described previously (Jenull et al., 2020). The *NAT1* selection marker was amplified from the plasmid pTS50 (Schwarzmüller et al., 2014). PCR products were gel purified and approximately 1 ng of each fragment was used for the fusion PCR reaction yielding in the final deletion construct, which was purified *via* ethanol precipitation. Transformation of *C. auris* with the *CDR1* deletion cassette was carried out as reported earlier (Reuß et al., 2004). Correct genomic integration of the deletion construct and the loss of the *CDR1* gene was verified by colony PCR (Tscherner et al., 2015). Oligos used in *CDR1* gene deletion are listed in **Supplementary Table 2**.

### Transcriptional Profiling Using RNA-Sequencing

For RNA-seq analysis, overnight-grown Candida cultures were inoculated into YPD (initial OD_600_ of 0.1) and grown at 37°C for 4h. Total RNA was purified using Genejet RNA purification kit (Thermo Scientific). Quality of RNA was assessed on a Bioanalyzer using the RNA6000 Nanochip (Agilent), mRNA was enriched using oligo(dT) beads (NEB) and subsequently, double-stranded cDNA libraries were generated by using the NEBNext Ultra Directional RNA Library Prep Kit for Illumina (NEB) according to the manufacturer’s instructions. The qualified libraries were subjected to Illumina sequencing with a 75 bp paired-read length at the Novogene (Novogene, USA) sequencing facility. Three biological replicates for each strain were sequenced.

Quality control of raw sequencing reads was done using fastQC v0.11.8 (Andrews, 2010). TrueSeq (Illumina) adapters were trimmed using cutadapt v1.18 (https://cutadapt.readthedocs.io/en/stable/; settings: -q 30) followed by read mapping onto the *C. auris* B11221 genome assembly (V1, NCBI RefSeq GCF_002775015.1) using NextGenMap v0.5.5 (Sedlazeck et al., 2013) (settings: -b). Optical read duplicates were removed using Picard tools (Broad Institute, https://broadinstitute.github.io/picard/, settings: MarkDuplicates REMOVE_SEQUENCING_DUPLICATES=true). Read counting was done using HTseq (Anders et al., 2014) in the union mode and the genomic annotation from *C. auris* B11221 (settings: -f bam -t gene -i ID). Differential gene expression analysis was done using pair-wise comparisons in edgeR (Robinson et al., 2009). The false discovery rate (FDR) represents p-values adjusted for multiple testing using the Benjamini-Hochberg procedure (Benjamini and Hochberg, 1995). Normalized read counts were extracted using the edgeR ‘cpm’ function and were used for principal component analysis (PCA) using the ‘prcomp’ function in R.

For further downstream analysis, *C. auris* genes detected in the RNA-seq analysis were aligned to *C. albicans* homologues using the BLAST+ tools (NCBI) using standard parameters. Protein sequences for *C. albicans* were retrieved from the Candida Genome Database (CGD) and for *C. auris* from NCBI. *C. albicans* protein sequences were used to create a BLAST database using the makeblastdb tool. Subsequently, all *C. auris* proteins were queried against this database using the blastp tool and standard parameters. Only hits with an E-value of < 0.001 and a query protein cover of > 50% were allowed to identify a BLAST hit as *C. auris* homologue. GO term enrichment analysis based on the *C. albicans* homologues was performed using the ‘enrichGO’ function from the clusterProfiler package (Yu et al., 2012). Only GO categories with a q-value < 0.05 were considered significant. The RNA-seq analysis results are presented in **Supplementary Table 3**, **Supplementary Table 5**.

### Fluorescein Diacetate Uptake Assay

The kinetics of FDA uptake was carried out essentially as described earlier (Shivarathri et al., 2020) with minor modifications. Briefly, *C. auris* strains were grown to the logarithmic growth phase at 37°C, washed twice in 1 ml of FDA buffer (50 mM HEPES, pH 7.0 and 0.5 mM 2-deoxy-D-glucose [all Sigma-Aldrich]), followed by the addition of 50 nM FDA (ThermoFisher). The kinetics of FDA uptake were recorded every 5 min with continuous shaking at 37°C using a H1 Synergy plate reader (Biotek) with excitation and emission wavelengths set to 485 and 535 nm, respectively. Data represent the arbitrary units (AU) of mean fluorescence intensity over time. The slope was calculated in GraphPad v6.01 (Prism).

### Assessment of Proteolytic Growth

Proteolytic growth of fungal strains was analyzed on solid YCB-BSA medium. Strains were grown overnight in YPD at 30°C with constant agitation (200 rpm), washed 2x with dH_2_O and finally resuspended in dH_2_O prior cell-counting on a CASY cell counter (Roche). Cells were diluted to 2.0 x 10^5^ cells/ml and 5 µl of this dilution were spotted onto a YCB-BSA plate. Colony growth and proteolytic halo formation was recorded after 3 days at 30°C.

## Results

### Intra-Clade Variation Among *C. auris* Clade I Isolates in Antifungal Susceptibility

**Figure 1:**
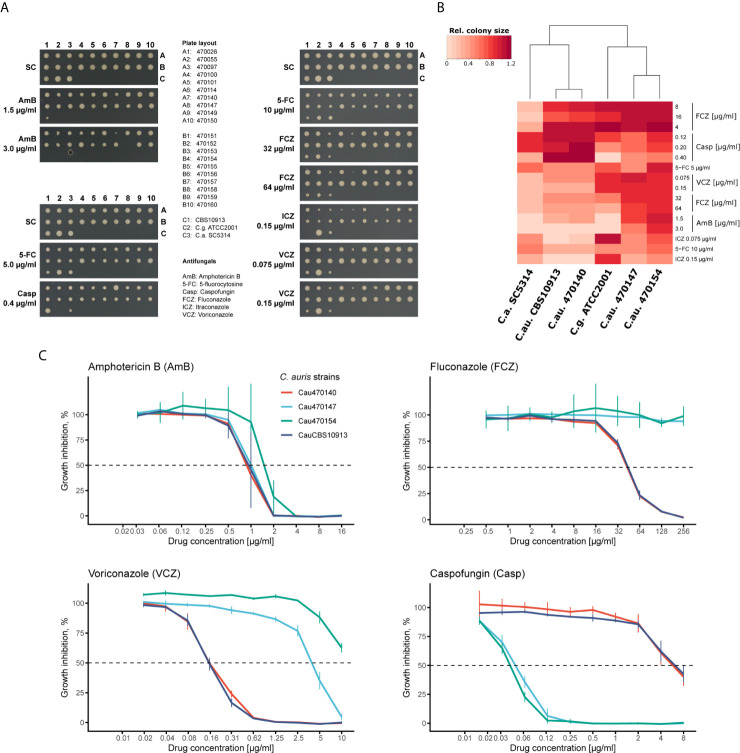
C. *auris* clinical isolates show variations in antifungal susceptibility. **(A)** Plate-based drug susceptibilities of clade-I C. *auris* clinical isolates. Overnight cultures in YPD medium were spotted on synthetic complete medium plates containing the indicated antifungals. Plates were imaged after 3 days incubation at 30°C. **(B)** Heatmap of screening results shown in A for the indicated strains. Colony sizes were quantified and normalized to control plates. Color codes indicate the relative colony size for the corresponding condition and represents the mean of two screening plates. **(C)** Confirmation of plate-based screening results using liquid growth inhibition assays. Cells were incubated in YPD medium with the indicated antifungal drugs at 30°C for 24 hours prior to OD_600_ measurement. Data represent mean +/- SD from 3 biological replicates.

Recent large-scale profiling of *C. auris* isolates from different clades demonstrated extensive genomic variations (Muñoz et al., 2018). However, even within one clade, antifungal susceptibilities can vary (Chowdhary et al., 2018; Chow et al., 2020). Thus, we further explored the intra-clade variation of clade I *C. auris* clinical isolates using a solid-medium screening approach. We tested fungal susceptibilities representative from all antifungal classes (azoles, echinocandins, polyenes and nucleoside analogues) and additionally included reference control strains such as *C. albicans* (SC5314), *C. glabrata* (ATCC2001) and the initial *C. auris* isolate from Japan (CBS10913, clade II; (Satoh et al., 2009)). As expected, *C. auris* isolates differed in their antifungal susceptibility profiles (**Figure 1A**, **Supplementary Figure 1**). For instance, isolate 470140 showed pronounced growth inhibition by AmB, itraconazole (ICZ) and a moderate growth inhibition by fluconazole (FCZ) or voriconazole (VCZ), while growth was unaffected by the presence of caspofungin (colony A7 in **Figure 1A**). By contrast, isolates 470147 and 470154 (colony A8 and B4, respectively in **Figure 1A**) were much less susceptible to all tested azoles, but showed reduced colony sizes upon caspofungin treatment when compared to isolate 470140 (**Figure 1A**). In line with this observation, clustering based on colony sizes upon antifungal treatment confirmed that isolates 470147 and 470154 clustered apart from isolate 470140 based on their susceptibility profiles (**Figure 1B**). This further highlights the seemingly opposing antifungal sensitivities between isolates 470140 and 470147 or 470154. We further validated the plate-based screening results for 470140, 470147 and 470154 using a liquid microbroth dilution assay. Again, we observed a striking difference in azole and caspofungin susceptibility between the shortlisted isolates, thus fully confirming the solid-media screening data (**Figure 1C**). Notably, the azole-resistant and caspofungin-sensitive isolates 470147 and 470154 displayed a divergent VCZ phenotype in the liquid microbroth dilution assay and the solid medium screening, with 470154 showing greater VCZ susceptibility than 470147 in the latter assay and *vice versa.* Given that most *C. auris* isolates are inherently FCZ-resistant, but echinocandin-sensitive (Chowdhary et al., 2018; Kordalewska et al., 2018), we chose those isolates for further characterization. Of note, the CBS10913 control isolate, previously reported to be echinocandin-sensitive (Muñoz et al., 2018), displayed only minor growth inhibition after caspofungin treatment in the experimental conditions used here. This discrepancy may come from different protocols employed for antifungal susceptibility testing.

### Transcriptional Profiles of *C. auris* With Distinct Antifungal Susceptibility Profiles

**Figure 2:**
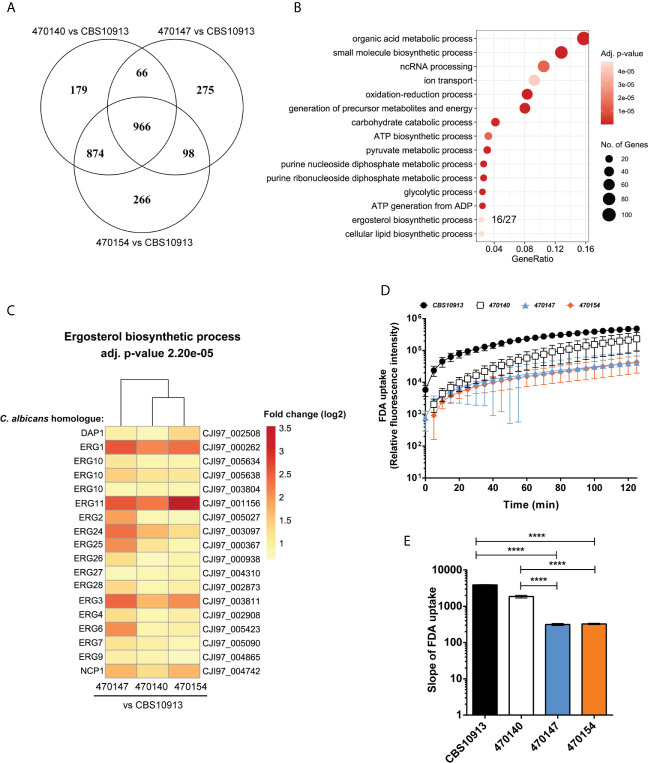
Transcriptome analysis of C. *auris* clinical isolates. **(A)** Genome-wide transcript levels were determined for C. *auris* clinical isolates using RNA-seq. The number of differentially expressed genes (FDR < 0.05, fold change > 1.5 or < -1.5) and commonly regulated genes of pairwise comparisons are visualized. **(B)** GO-term enrichment analysis of commonly differentially expressed genes in clade I isolates relative to the CBS10913 type strain (intersection of the Venn diagram in panel **(A)**. The GeneRatio denotes the number or genes enriched in the depicted GO-term relative to the total number of genes associated with this GO-term. **(C)** Heatmap depicting genes enriched in the GO-term “ergosterol biosynthetic process” from panel **(B)** The color code indicates the fold-changes (log2) in gene expression. Gene names on the left side refer to C. *albicans* homologues as determined by BLASTp and labels on the right side depict C *auris* gene IDs. **(D, E)** Kinetics **(D)** and the slope **(E)** of FDA uptake by C. *auris* strains as indicated. Data represent mean + SD from 4 biological replicates. ****P < 0.0001 with one-way ANOVA and Tukey’s multiple comparison test.

Distinct transcriptional programs often link genomic and epigenetic variations, and dictate phenotypic diversity (Halme et al., 2004; Richards, 2006). To identify molecular mechanisms conferring inter- and intra-clade variations in a broader sense, we determined genome-wide transcriptional profiles of clade I isolates 470147, 470154 (both azole-resistant), 470140 (azole-sensitive), as well as the clade II CBS10913 strain during standard growth in rich medium. First, we examined inter-clade variations and compared the number of differentially expressed genes (DEGs) between clade I isolates with CBS10913. We found a large set of genes at least 1.5-fold differentially expressed (roughly 600-1100 genes, depending on the comparison, **Table 1**), which may arise from vast inter-clade genomic variations (Muñoz et al., 2018). Accordingly, 966 genes (FDR < 0.05 and > 1.5-fold change) were commonly deregulated between all clade I isolates and CBS10913 (**Figure 2A**, **Supplementary Table 4**). Strikingly, gene ontology analysis revealed that almost 60% of genes associated with ergosterol biosynthetic processes (16 out of 27 annotated genes) were significantly enriched among the gene set commonly differentially expressed between clade I isolates and the CBS10913 strain (**Figure 2B**, **Supplementary Table 4**). Remarkably, this gene set with enhanced expression in clade I isolates contained 14 out of 21 ergosterol biosynthesis genes (**Figure 2C**, **Supplementary Figure 2**). Interestingly, the key transcriptional regulator of ergosterol biosynthesis genes *UPC2* (Silver et al., 2004) was additionally upregulated in all clade I strains with respect to the CBS10913 isolates (**Table 2**). To test whether this altered transcriptional control of ergosterol biosynthesis genes translates into altered cell membrane properties, we performed a fluorescein diacetate (FDA) uptake assay. FDA enters only *via* passive diffusion and thus, is solely a function of non-protein membrane permeability (Breeuwer et al., 1995). Indeed, we found that membrane permeability was 5 to 10-fold decreased in clade I isolates when compared to the clade II CBS10913 strain (**Figure 2D**, **E**), which functionally validates the detected transcriptional deregulation of ergosterol biosynthesis. Moreover, FDA uptake by azole-resistant 470147 and 470154 was roughly 2-fold lower than in the azole-sensitive 470140 strain (**Figure 2D**, **E**), which could explain differential antifungal susceptibilities profiles.

**Table 1:** Number of differentially expressed genes (RNA-seq).

Comparison	Up	Down
**470140 vs CBS10913**	955	1135
**470147 vs CBS10913**	624	787
**470154 vs CBS10913**	998	1214
**470154 vs 470140**	106	195

Cut-off: log2 fold change 0.58 (~ 1.5-fold change) & false discovery rate (FDR) < 0.05.

**Table 2:** Transcriptional regulation of *UPC2* (RNA-seq).

*UPC2* regulation		
Comparison	Fold change (log2)	FDR
**470140 vs CBS10913**	0.49	7.56298E-07
**470147 vs CBS10913**	1.36	1.43291E-42
**470154 vs CBS10913**	0.54	6.86098E-08

FDR, false discovery rate

### Minor Transcriptional Variations Suffice to Establish Distinct Antifungal Susceptibilities

**Figure 3:**
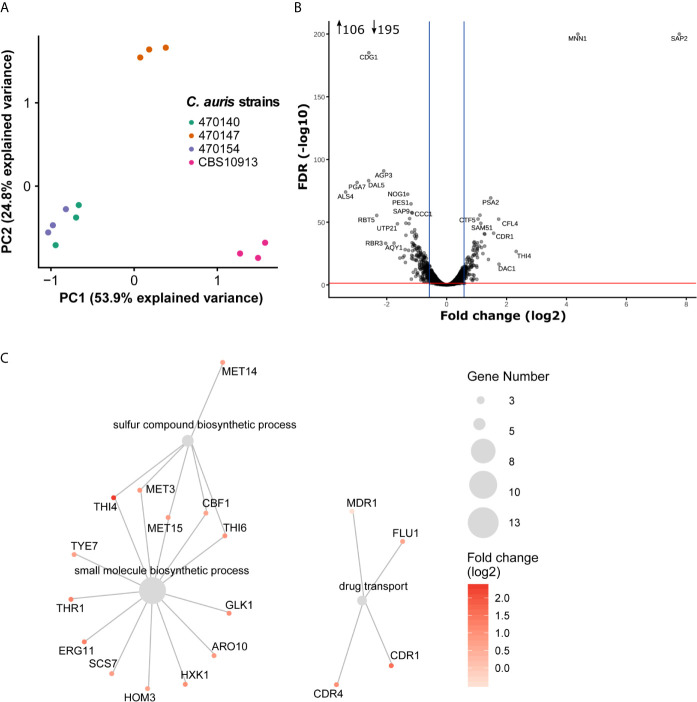
Minor transcriptional variation drives divergent antifungal susceptibilities. **(A)** Principal component analysis result using normalized RNA-seq read counts from three biological replicates per strain. The two clinical isolates 470140 and 470154 show high similarity in their gene expression profile and were used for further pairwise comparison. **(B)** Pairwise differential expression analysis of 470154 vs 470140. Fold-change (log2) in 470154 vs 470140 is plotted against the FDR. Top differentially expressed genes are labeled based on their homologues found in C. *albicans*. Blue lines indicate 1.5-fold change and the red line marks a FDR of 0.05. Number inserts depict differentially expressed genes (FDR < 0.05 and log2 fold change > 0.58 [~ 1.5-fold change] or < -0.58 [~ -1.5-fold change]) **(C)** GO term enrichment analysis of genes up-regulated (1.5-fold change with FDR < 0.05) in 470154 vs 470140. Grey dots represent significantly enriched GO terms and colored dots with lines show genes belonging to the corresponding GO term. Dot size indicates the number of genes within a GO term and the color code reflects expression changes in 470154 vs 470140.

Inter-clade genomic variation is approximately 17-fold higher than among isolates from the same clade (Muñoz et al., 2018). This may be accompanied by large transcriptional re-wiring, which might make identification of key pathways mediating phenotypic identities challenging. Therefore, we aimed to pinpoint intra-clade transcriptional variations underlying specific antifungal susceptibility profiles, which are not biased by large inter-clade genomic variations. As shown above, the isolates 470140 and 470154 show opposing sensitivities to azoles and caspofungin, while isolate 470147 phenocopies 470154 (**Figures 1B, C**). Yet, isolates 470154 and 470140 share exclusively 874 DEGs when compared to CBS10913, whereas 470154 and 470147 only shared a common DEGs set of 98 genes (**Figure 2A**). This was further reflected in a principal component analysis (PCA), where CBS10913 was separated from the other isolates by the first PC, explaining roughly 54% of variance among the samples (**Figure 3A**). In contrast, isolate 470154 and 470140 appeared highly similar in their transcriptional profile as they clustered together, despite their opposing azole and caspofungin susceptibilities. Therefore, we performed a pairwise comparison of those two isolates and found a limited set of 301 genes at least 1.5-fold differentially expressed (**Table 1**, **Figure 3B**). The top downregulated genes encompassed several cell wall-associated proteins such as homologues of the *C. albicans ALS4* and *PGA7*, encoding for an adhesin (Hoyer et al., 1998) and a GPI-anchored cell wall protein (Sorgo et al., 2013), respectively (**Figure 3B**). In addition, homologues of *C. albicans SAP2*, encoding a secreted aspartic protease (Hube et al., 1994), and *MNN1*, encoding for a mannosyltransferase (Bates et al., 2013), were strikingly upregulated in isolate 470154 when compared to 470140 (**Figure 3B**). As 470154 and 470140 diverge greatly in their azole susceptibilities, we next aimed to identify genes associated with azole responses among DEGs in 470154 vs 470140.

Fungal azole resistance is often mediated by an ectopic expression of drug exporters such as ATP-Binding Cassette (ABC) and Major Facilitator Superfamily (MFS) transporters (Prasad et al., 2019). Indeed, we found the homologue of the *C. albicans* ABC transporter *CDR1* (Kim et al., 2019) upregulated in the azole-resistant 470154 isolate when compared to 470140 (**Figure 3B**). Since overexpression of additional antifungal drug targets has been observed in resistant isolates (Muñoz et al., 2018; Zamith-Miranda et al., 2019), we performed gene ontology (GO) analysis of genes upregulated (FDR < 0.05 and 1.5-fold change, **Supplementary Table 6**) in the azole resistant isolate 470154 with respect to the azole sensitive isolate 470140. Indeed, processes involving drug transport were significantly enriched with upregulated genes in the azole-resistant 470154 isolate (**Supplementary Table 6**). Those included homologues of the *C. albicans* MFS transporters *MDR1* (Pasrija et al., 2007) and *FLU1* (Calabrese et al., 2000), as well as the ABC transporters *CDR1* (Prasad et al., 1995) and *CDR4* (Franz et al., 1998). Notably, despite the general upregulation of ergosterol biosynthesis genes in clade I isolates with respect to clade II CBS10913 (**Figure 2C**), expression of the key azole target gene *ERG11*, encoding the lanosterol 14-α-demethylase (Hitchcock et al., 1990), was additionally increased in isolate 470154 when compared to 470140 (**Figure 3C**). These transcriptional aberrations might cooperate to prime distinct cellular properties such as cell permeability and azole uptake or export (**Figure 2D**) and thus explain distinct antifungal susceptibility profiles (**Figure 1B**).

### Transcriptional Aberrations of Key Players Determine Fungal Phenotypic Identities

**Figure 4:**
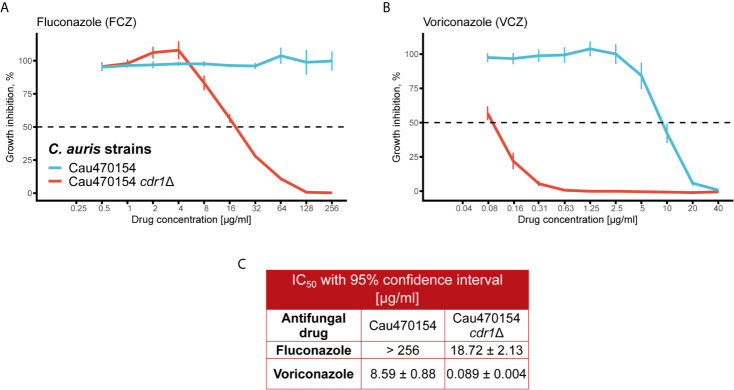
Deletion of *CDR1* abolishes azole resistance in C. *auris*. **(A)** The C. *albicans CDR1* homologue was deleted in 470154 and fluconazole susceptibility was quantified using a liquid growth inhibition assay. Cells were incubated with the indicated antifungal concentrations at 30°C for 24 hours prior to OD_600_ measurement. Fluconazole resistance is abolished upon deletion of *CDR1*. **(B)** Lack of *CDR1* renders 470154 susceptible to voriconazole. Cells were treated as described in **(A)**. **(C)** 50% inhibitory concentrations (IC_50_) for antifungals tested in A-B are listed. **(A, B)** Data are shown as mean +/- SD from 4 biological replicates.

To decipher true effectors mediating phenotypic and MDR variation, we next validated differentially expressed genes between isolates 470154 and 470140. We first focused on genes modulating fungal azole sensitivity, such as drug exporters (Prasad et al., 2019). As mentioned above, the ABC transporter *CDR1* was among the top upregulated genes in the azole-resistant 470154 when compared to the azole sensitive 470140 isolate (**Figure 3B**). To confirm the relevance of *CDR1* overexpression in this specific context, we deleted *CDR1* in the 470154 background and assessed the susceptibilities to FCZ and VCZ using a microbroth dilution assay in rich medium. As already observed in earlier studies for other clinical isolates (Kim et al., 2019; Rybak et al., 2019), genetic ablation of *CDR1* essentially restored azole sensitivity of strain 470154 (**Figures 4A, B**). This was reflected in a more than 10-fold reduction of IC_50_ values for both FCZ and VCZ (**Figure 4C**). These data further highlight the global biological relevance of Cdr1 in *C. auris* azole resistance and fully confirm our RNA-seq data.

**Figure 5:**
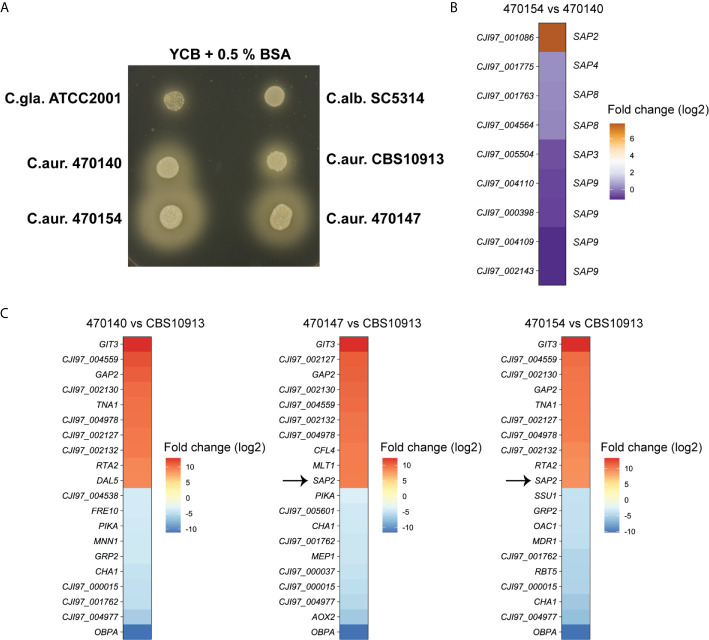
Overexpression of C. *auris SAP2* increases proteolytic growth. **(A)** The indicated strains were spotted on YCB medium supplemented with 0.5% BSA and incubated at 30°C for 3 days. Turbid zones around the colony are indicative of proteolytic activity. C.gla., C. *glabrata*; C.alb., C. *albicans*, C.aur., *C.auris*. **(B)** Heatmap depicting the log2-fold changes in expression of C. *auris* genes, homologous to C. *albicans* secreted aspartic proteases, in strain 470154 vs 470140. Box labels on the left side show C. *auris* gene IDs (C. *auris* B11221), labels on the right side depict the corresponding C. *albicans* homologue based on BLASTp search results. **(C)** Heatmap of the top 10 up- and downregulated genes of the indicated comparison. Notably, *CJI97_002130, CJI97_001762* and *CJI97_004977* showed the greatest homology to C. *albicans SIT1* based on BLASTp. The color code represent the log2 fold changes in gene expression.

Furthermore, another top-upregulated gene between 470154 and 470140, *SAP2* (**Figure 3B**), was also subjected to biological validation. The *C. albicans* genome encodes for at least 10 different secreted aspartic proteases (SAPs) (Naglik et al., 2003). Sap2 facilitates nitrogen assimilation when protein serves as the major nitrogen source and its expression is repressed in the presence of favorable nitrogen sources (Hube et al., 1994; Martínez and Ljungdahl, 2005). To test whether elevated *SAP2* expression in 470154 cells correlates with increased fungal proteolytic activity, we assessed colony behavior of *C. auris* isolates on YCB medium supplemented with BSA as the major nitrogen source. Indeed, 470154 displayed a greater halo around the colonies, indicating strongly increased extracellular proteolytic BSA degradation (Bernardo et al., 2008), when compared to the *SAP2*-low expressing isolate 470140 (**Figure 5A**). To correlate the increased proteolytic activity of 470154 solely with *SAP2* overexpression, we further compared expression levels of additional SAP family members in 470154 and 470140. We detected 9 putative SAPs expressed in the *C. auris* RNA-seq data set, which were homologous of *C. albicans SAP2*, *SAP3*, *SAP4*, *SAP8* and *SAP9*. Interestingly enough, only *SAP2* was significantly upregulated in 470154 cells (**Figure 5B**), thus functionally validating the RNA-seq data. Notably, isolate 470147, which phenocopies 470154 in terms of antifungal susceptibility profiles, displayed increased proteolytic activity when compared to isolate 470140 or the CBS10913 strain (**Figure 5A**). This is in line with the specific upregulation of *SAP2* by isolates 470154 and 470147, but not 470140, when compared to the CBS10913 type strain (**Figure 5C**). In summary, transcriptional profiling during fungal steady state growth can robustly uncover principle phenotypic variation of *C. auris* clinical isolates at the inter- and intra-clade levels.

## Discussion

*C. auris* shows vast intra-species genetic diversities, often reflected in distinct phenotypic behaviors including antifungal susceptibilities. While pioneering studies provided new insights into the genomic variability (Sharma et al., 2016; Lockhart et al., 2017; Muñoz et al., 2018), mechanistic studies on how those genomic make-ups translate into intra-clade and inter-clade transcriptional and phenotypic variability are required to better understand the emergence, global spread and pathophysiology of this pathogen. Here, we present transcriptional signatures of *C. auris* isolates with distinct antifungal susceptibility profiles at both the intra- and inter-clade levels. We found that clade I isolates differ strikingly from the clade II type isolate CBS10913 in their gene expression profiles during steady state growth. This may reflect the genetic diversity between clade I and clade II isolates (Chow et al., 2020). Depending on genomic locations of genetic polymorphisms, they may affect multiple steps of transcriptional control, such as the recruitment of transcriptional regulators or even mRNA stability. This may impact the degree of transcriptional and phenotypic diversity driven by even minor genetic variations (Williams et al., 2007; Brion et al., 2015). One of the most striking transcriptional alterations affected genes of the ergosterol biosynthesis pathway. We found that all ergosterol biosynthesis genes acting downstream of mevalonate were upregulated in azole-resistant isolates. This suggests a bias towards late ergosterol precursors within the biosynthesis pathway. The transcription factor Upc2 is a sterol-sensing regulator (Yang et al., 2015) and critical for the regulation of ergosterol biosynthesis (Silver et al., 2004). Indeed, *UPC2* was upregulated in clade I isolates when compared to CBS10913. This may also explain the broad dysregulation of most genes in facilitating ergosterol biosynthesis. The ergosterol content determines fundamental membrane properties such as rigidity and permeability that control non-protein-mediated drug uptake by diffusion (Abe and Hiraki, 2009). In line with this, clade I isolates display decreased FDA uptake, suggesting reduced membrane permeability when compared to the CBS10913 strain. Notably, FDA uptake is impaired in azole-resistant clade I isolates (470147 and 470154) when compared to the azole-sensitive strain (470140). An altered membrane permeability might thus partially explain divergent azole susceptibilities of isolates, even if cellular azole uptake not only relies on passive diffusion (Mansfield et al., 2010; Esquivel et al., 2015). Nevertheless, the transcriptional signatures of ergosterol biosynthesis reflect principle phenotypes related to fungal membrane permeability.

The bulk transcriptional dysregulation of the ergosterol biosynthesis pathway is most likely not the only driver for divergent azole-susceptibilities. Instead, several additional mechanisms such as *ERG11* mutations (Chowdhary et al., 2018), chromosomal aberrations (Bing et al., 2020) or upregulation of drug efflux pumps and their key transcriptional regulator *TAC1B* (Wasi et al., 2019; Rybak et al., 2020) have also been implicated in *C. auris* azole resistance. For instance, increased expression of the ABC transporter Cdr1 was detected in azole resistance isolates (Kim et al., 2019; Rybak et al., 2019; Zamith-Miranda et al., 2019) (Rybak et al., 2020), as originally reported for the *C. albicans* orthologue (Sanglard et al., 1995). In line with these reports, *CDR1* transcript levels are increased in the azole-resistant 470154 isolate when compared to the azole-sensitive 470140 strain. Remarkably, genetic ablation of *CDR1* strongly enhanced azole susceptibility of 470154. These data hint that a selective reversal or pharmacological inhibition of *CDR1* in azole-resistant *C. auris* strains could offer therapeutic benefits in clinical settings, although this has remained a highly controversial topic in the MDR field (Holmes et al., 2016). Besides divergent azole susceptibilities of isolate 470154 and 470140, those strains also displayed opposing caspofungin sensitivities, with 470154 being more susceptible and 470140 showing increased tolerance. Despite the well-known link of Cdr1 in azole resistance (Sanglard et al., 1995), Cdr1 seems to play no significant role in echinocandin susceptibility (Niimi et al., 2006). Of note, in *C. albicans*, ectopic overexpression of Cdr2, but not Cdr1, promotes caspofungin resistance in clinical isolates, although it has not been established if this is a direct or indirect effect of Cdr2 gene dosage (Schuetzer-Muehlbauer et al., 2003). In general, the pairwise comparison of transcriptional profiles at the clade level revealed that minor transcriptional changes suffice to drive divergent antifungal susceptibilities. Similarly, a previous study found limited differences in the metabolic, lipidomic and proteomic profiles between two independent *C. auris* isolates displaying distinct antifungal sensitives (Zamith-Miranda et al., 2019). From a mechanistic point of view, a limited set of differentially expressed genes facilitates the identification of true effector pathways driving phenotypic variability. For instance, based on transcriptional profiles, we decipher divergent proteolytic growth traits and functionally verify the *C. auris* homologue *SAP2*, which is one of the most highly regulated genes in resistant isolates. Given that SAPs are implicated in fungal adhesion and tissue invasion (Naglik et al., 2003), we speculate that *SAP2* deregulation may also contribute to or is even critically determining attachment to biotic surfaces such as human skin. This can now be tested with appropriate skin models of fungal colonization (Huang et al., 2020; Rakita et al., 2020). Importantly, the pronounced adhesion to skin not only is a key trait of *C. auris* pathophysiology, it has been recognized as the most important route of person-to-person transmission in hospital settings, thus contributing to the global spreading and pandemic potential of *C. auris* (Forsberg et al., 2019).

Our data demonstrate that transcriptional signatures facilitate the prediction of intra-clade phenotypic variability and associated virulence traits. Of note, as we subjected a limited set of *C. auris* isolates to transcriptional profiling, the resulting predictions may not cover the entire diversity of intra- or interclade susceptibility phenotypes, as distinct genomic make-ups, even when only minute, may cause massive transcriptional alterations. To what extent those transcriptional profiles correlate with genomic diversity or genome dynamics remains to be investigated. Pathogen phenotypes are not solely driven by DNA sequence, but are a result of complex genetic and epigenetic interactions (Richards, 2006; Skelly et al., 2013), with the latter one facilitating fast and also heritable phenotypic adaptations (De Fine Licht, 2018). Indeed, the ability for rapid adaptations become essential for pathogen survival upon host immune defense encounters (Alves et al., 2020). Hence, it would be further interesting to analyze in more detail the link between epigenetic changes and intra-clade diversification in *C. auris*. This may be further driven by repeated host exposure as *C. auris* is readily transmitted between patients of distinct immunological backgrounds (Forsberg et al., 2019). Moreover, adaptive phenotypic alterations have been observed for re-isolated *C. albicans* (Pande et al., 2013; Forche et al., 2018) and *C. auris* (Yue et al., 2018) from infected mice.

In summary, we show here in principle that comparative transcriptional profiling of *C. auris* clinical isolates enables the prediction and diagnosis of phenotypic variation such as MDR traits. Transcriptional profiling of larger sets of clinical isolates, and the integration with genomic and phenotypic data, are now feasible owing to the stunning advances in deep-sequencing technologies. This approach may facilitate the robust prediction of fungal virulence and MDR phenotypes, which will advance our understanding of *C.auris* biology and species diversification.

## Data Availability Statement

Scripts for the primary RNA-seq analysis are deposited on github: https://github.com/tschemic/RNAseq_analysis_Cauris. Raw RNA-seq data have been deposited at the Gene Expression Omnibus (GEO) under the accession number GSE165762.

## Author Contributions

Conceptualization and experimental design: SJ, MT, KK. Performed experiments: NK, RS, SJ, AS, MC, AP. Analyzed data: NK, MT, SJ. Wrote the manuscript: SJ, KK. Acquired funding: KK, NC. All authors contributed to the article and approved the submitted version.

## Funding

This work was funded by a grant from the Austrian Science Fund FWF (*ChromFunVir*; P-32582). NK was supported by a Vienna Biocenter Summer School studentship. In addition, support came in part from a grant funded by the National Institute of Health to NC and KK (R01AI124499). MT was supported by an Erwin Schroedinger Return Fellowship (J3835) of the Austrian Science Fund FWF.

## Conflict of Interest

The authors declare that the research was conducted in the absence of any commercial or financial relationships that could be construed as a potential conflict of interest.

## References

[B1] Abe F. Hiraki T. (2009). Mechanistic role of ergosterol in membrane rigidity and cycloheximide resistance in *Saccharomyces cerevisiae* . Biochim. Biophys. Acta Biomembr 1788, 743–752. 10.1016/j.bbamem.2008.12.002 19118519

[B2] Alves R. Barata-Antunes C. Casal M. Brown A. J. P. Van Dijck P. Paiva S. (2020). Adapting to survive: How Candida overcomes host-imposed constraints during human colonization. PloS Pathog. 16, e1008478. 10.1371/journal.ppat.1008478 32437438 PMC7241708

[B3] Anders S. Pyl P. T. Huber W. (2014). HTSeq—a Python framework to work with high-throughput sequencing data. Bioinformatics 31, 166–169. 10.1093/bioinformatics/btu638 25260700 PMC4287950

[B4] Andrews S. (2010). FastQC: A quality control tool for high throughput sequence data. Available at: http://www.bioinformatics.babraham.ac.uk/projects/fastqc/.

[B5] Arendrup M. C. Prakash A. Meletiadis J. Sharma C. Chowdhary A. (2017). Comparison of EUCAST and CLSI Reference Microdilution MICs of Eight Antifungal Compounds for *Candida auris* and Associated Tentative Epidemiological Cutof. Antimicrob. Agents Chemother. 61, e00485–e00417. 10.1128/AAC.00485-17 PMC5444165 28416539

[B6] Bates S. Hall R. A. Cheetham J. Netea M. G. MacCallum D. M. Brown A. J. P.. (2013). Role of the *Candida albicans* MNN1 gene family in cell wall structure and virulence. BMC Res. 6, 294. 10.1186/1756-0500-6-294 PMC3750861 23886038

[B7] Benjamini Y. Hochberg,. Y. (1995). Controlling the False Discovery Rate: A Practical and Powerful Approach to Multiple Testing. J. R. Stat. Soc Ser. 57, 289–300.

[B8] Berman J. Krysan D. J. (2020). Drug resistance and tolerance in fungi. Nat. Rev. Microbiol. 18, 319–331. 10.1038/s41579-019-0322-2 32047294 PMC7231573

[B9] Bernardo S. M. Khalique Z. Kot J. Jones J. K. Lee S. A. (2008). *Candida albicans* VPS1 contributes to protease secretion, filamentation, and biofilm formation. Fungal Genet. Biol. 45, 861–877. 10.1016/j.fgb.2008.01.001 18296085 PMC2729247

[B10] Bing J. Hu T. Zheng Q. Muñoz J. F. Cuomo C. A. Huang G. (2020). Experimental Evolution Identifies Adaptive Aneuploidy as a Mechanism of Fluconazole Resistance in *Candida auris* . Antimicrob. Agents Chemother. 65, e01466–e01420. 10.1128/AAC.01466-20 33077664 PMC7927865

[B11] Blair J. M. A. Webber M. A. Baylay A. J. Ogbolu D. O. Piddock L. J. V. (2015). Molecular mechanisms of antibiotic resistance. Nat. Rev. Microbiol. 13, 42–51. 10.1038/nrmicro3380 25435309

[B12] Breeuwer P. Drocourt J. L. Bunschoten N. Zwietering M. H. Rombouts F. M. Abee T. (1995). Characterization of uptake and hydrolysis of fluorescein diacetate and carboxyfluorescein diacetate by intracellular esterases in *Saccharomyces cerevisiae*, which result in accumulation of fluorescent product. Appl. Environ. Microbiol. 61, 1614–1619. 10.1128/AEM.61.4.1614-1619.1995 7747975 PMC167417

[B13] Brion C. Pflieger D. Friedrich A. Schacherer J. (2015). Evolution of intraspecific transcriptomic landscapes in yeasts. Nucleic Acids Res. 43, 4558–4568. 10.1093/nar/gkv363 25897111 PMC4482089

[B14] Bruno M. Kersten S. Bain J. M. Jaeger M. Rosati D. Kruppa M. D.. (2020). Transcriptional and functional insights into the host immune response against the emerging fungal pathogen Candida auris. Nat. Microbiol. 5, 1516–1531. 10.1038/s41564-020-0780-3 32839538 PMC9204844

[B15] Burton,. A. Torres-Padilla M.-E. (2014). Chromatin dynamics in the regulation of cell fate allocation during early embryogenesis. Nat. Rev. Mol. Cell Biol. 15, 723–734. 10.1038/nrm3885 25303116

[B16] Calabrese D. Bille J. Sanglard D. (2000). A novel multidrug efflux transporter gene of the major facilitator superfamily from *Candida albicans* (FLU1) conferring resistance to fluconazole. Microbiology 146, 2743–2754. 10.1099/00221287-146-11-2743 11065353

[B17] Chen J. Tian S. Han X. Chu Y. Wang Q. Zhou B.. (2020). Is the superbug fungus really so scary? A systematic review and meta-analysis of global epidemiology and mortality of *Candida auris* . BMC Infect. Dis. 20, 1–10. 10.1186/s12879-020-05543-0 PMC7656719 33176724

[B18] Chow N. A. Gade L. Tsay S. V. Forsberg K. Greenko J. A. Southwick K. L.. (2018). Multiple introductions and subsequent transmission of multidrug-resistant *Candida auris* in the USA: a molecular epidemiological survey. Lancet Infect. Dis. 18, 1377–1384. 10.1016/S1473-3099(18)30597-8 30293877 PMC6556114

[B19] Chow N. A. de Groot T. Badali H. Abastabar M. Chiller T. M. Meis J. F. (2019). Potential Fifth Clade of *Candida auris*, Ira Emerg. Infect. Dis. 25, 1780–1781. 10.3201/eid2509.190686 PMC6711235 31310230

[B20] Chow N. A. Muñoz J. F. Gade L. Berkow E. L. Li X. Welsh R. M.. (2020). Tracing the Evolutionary History and Global Expansion of *Candida auris* Using Population Genomic Analyses. MBio 11, e03364–e03319. 10.1128/mBio.03364-19 32345637 PMC7188998

[B21] Chowdhary A. Sharma C. Meis J. F. (2017). *Candida auris*: A rapidly emerging cause of hospital-acquired multidrug-resistant fungal infections globally. PloS Pathog. 13, 1–10. 10.1371/journal.ppat.1006290 PMC5436850 28542486

[B22] Chowdhary A. Prakash A. Sharma C. Kordalewska M. Kumar A. Sarma S.. (2018). A multicentre study of antifungal susceptibility patterns among 350 *Candida auris* isolate -17) in India: Role of the ERG11 and FKS1 genes in azole and echinocandin resistance. J. Antimicrob. Chemother. 73, 891–899. 10.1093/jac/dkx480 29325167

[B23] Cloeckaert A. Kuchler K. (2020). Grand Challenges in Infectious Diseases: Are We Prepared for Worst-Case Scenarios? Front. Microbiol. 11, 613383. 10.3389/fmicb.2020.613383 33329504 PMC7734098

[B24] De Fine Licht H. H. (2018). Does pathogen plasticity facilitate host shifts? PloS Pathog. 14, e1006961. 10.1371/journal.ppat.1006961 29723278 PMC5933697

[B25] Du H. Bing J. Hu T. Ennis C. L. Nobile C. J. Huang G. (2020). *Candida auris*: Epidemiology, biology, antifungal resistance, and virulence. PloS Pathog. 16, 1–18. 10.1371/journal.ppat.1008921 PMC7581363 33091071

[B26] Esquivel B. D. Smith A. R. Zavrel M. White T. C. (2015). Azole Drug Import into the Pathogenic Fungus Aspergillus fumigatus;. Antimicrob. Agents Chemother. 59, 3390–3398. 10.1128/AAC.05003-14 25824209 PMC4432157

[B27] Forche A. Cromie G. Gerstein A. C. Solis N. V. Pisithkul T. Srifa W.. (2018). Rapid Phenotypic and Genotypic Diversification After Exposure to the Oral Host Niche in *Candida albicans* . Genetics 209, 725–741. 10.1534/genetics.118.301019 29724862 PMC6028260

[B28] Forsberg K. Woodworth K. Walters M. Berkow E. L. Jackson B. Chiller T.. (2019). *Candida auris*: The recent emergence of a multidrug-resistant fungal pathogen. Med. Mycol 57, 1–12. 10.1093/mmy/myy054 30085270

[B29] Franz R. Michel S. Morschhäuser J. (1998). A fourth gene from the *Candida albicans* CDR family of ABC transporters. Gene 220, 91–98. 10.1016/S0378-1119(98)00412-0 9767132

[B30] Gómez-Díaz E. Jordà M. Peinado M. A. Rivero A. (2012). Epigenetics of Host-Pathogen Interactions: The Road Ahead and the Road Behind. PloS Pathog. 8, e1003007. 10.1371/journal.ppat.1003007 23209403 PMC3510240

[B31] Halme A. Bumgarner S. Styles C. Fink G. R. (2004). Genetic and epigenetic regulation of the FLO gene family generates cell-surface variation in yeast. Cell 116, 405–415. 10.1016/S0092-8674(04)00118-7 15016375

[B32] Hitchcock C. A. Dickinson K. Brown S. B. Evans E. G. Adams D. J. (1990). Interaction of azole antifungal antibiotics with cytochrome P-450-dependent 14 alpha-sterol demethylase purified from *Candida albicans* . Biochem. J. 266, 475–480. 10.1042/bj2660475 2180400 PMC1131156

[B33] Holmes A. R. Cardno T. S. Strouse J. J. Ivnitski-Steele I. Keniya M. V. Lackovic K.. (2016). Targeting efflux pumps to overcome antifungal drug resistance. Future Med. Chem. 8, 1485–1501. 10.4155/fmc-2016-0050 27463566 PMC5827819

[B34] Horton M. V. Johnson C. J. Kernien J. F. Patel T. D. Lam B. C. Cheong J. Z. A.. (2020). *Candida auris* Forms High-Burden Biofilms in Skin Niche Conditions and on Porcine Skin. MSphere 5, 1–8. 10.1128/mSphere.00910-19 PMC6977180 31969479

[B35] Hoyer L. L. Payne T. L. Hecht J. E. (1998). Identification of *Candida albicans* ALS2 and ALS4 and localization of als proteins to the fungal cell surface. J. Bacteriol 180, 5334–5343. 10.1128/JB.180.20.5334-5343.1998 9765564 PMC107581

[B36] Huang M. Y. Woolford C. A. May G. McManus C. J. Mitchell A. P. (2019). Circuit diversification in a biofilm regulatory network. PloS Pathog. 15, e1007787. 10.1371/journal.ppat.1007787 31116789 PMC6530872

[B37] Huang X. Hurabielle C. Drummond R. A. Bouladoux N. Desai J. V. Sim C. K.. (2020). Murine model of colonization with fungal pathogen *Candida auris* to explore skin tropism, host risk factors and therapeutic strategies. Cell Host Microbe 29, 210–221.e6. 10.1016/j.chom.2020.12.002 33385336 PMC7878403

[B38] Hube B. Monod M. Schofield D. A. Brown A. J. P. Gow N. A. R. (1994). Expression of seven members of the gene family encoding secretory aspartyl proteinases in *Candida albicans* . Mol. Microbiol. 14, 87–99. 10.1111/j.1365-2958.1994.tb01269.x 7830564

[B39] Jenull S. Tscherner M. Mair T. Kuchler K. (2020). ATAC-Seq Identifies Chromatin Landscapes Linked to the Regulation of Oxidative Stress in the Human Fungal Pathogen *Candida albicans* . J. Fungi 6, 182. 10.3390/jof6030182 PMC7559329 32967096

[B40] Kaiser C. Michaelis S. Mitchell A. (1994). “Methods in Yeast Genetics,” in A Laboratory Course Manual (New York: Cold Spring Harbor Laboratory Press).

[B41] Kathuria S. Singh P. K. Sharma C. Prakash A. Masih A. Kumar A.. (2015). Multidrug-Resistant *Candida auris* Misidentified as *Candida haemulonii*: Characterization by Matrix-Assisted Laser Desorption Ionization-Time of Flight Mass Spectrometry and DNA Sequencing and Its Antifungal Susceptibility Profile Variability by Vitek 2, CL. J. Clin. Microbiol. 53, 1823–1830. 10.1128/JCM.00367-15 25809970 PMC4432077

[B42] Kim S. H. Iyer K. R. Pardeshi L. Muñoz J. F. Robbins N. Cuomo C. A.. (2019). Genetic Analysis of *Candida auris* Implicates Hsp90 in Morphogenesis and Azole Tolerance and Cdr1 in Azole Resistance. MBio 10, e02529–e02518. 10.1128/mBio.02529-18 PMC6355988 30696744

[B43] Kordalewska M. Perlin D. S. (2019). Identification of Drug Resistant Candida auris. Front. Microbiol. 10, 1918. 10.3389/fmicb.2019.01918 31481947 PMC6710336

[B44] Kordalewska M. Lee A. Park S. Berrio I. Chowdhary A. Zhao Y.. (2018). Understanding Echinocandin Resistance in the Emerging Pathogen *Candida auris* . Antimicrob. Agents Chemother. 62, e00238–e00218. 10.1128/AAC.00238-18 29632013 PMC5971591

[B45] Lappalainen T. Greally J. M. (2017). Associating cellular epigenetic models with human phenotypes. Nat. Rev. Genet. 18, 441–451. 10.1038/nrg.2017.32 28555657

[B46] Lockhart S. R. Etienne K. A. Vallabhaneni S. Farooqi J. Chowdhary A. Govender N. P.. (2017). Simultaneous emergence of multidrug-resistant *Candida auris* on 3 continents confirmed by whole-genome sequencing and epidemiological analyses. Clin. Infect. Dis. 64, 134–140. 10.1093/cid/ciw691 27988485 PMC5215215

[B47] Mansfield B. E. Oltean H. N. Oliver B. G. Hoot S. J. Leyde S. E. Hedstrom L.. (2010). Azole drugs are imported by facilitated diffusion in *Candida albicans* and other pathogenic fungi. PloS Pathog. 6, e1001126–e1001126. 10.1371/journal.ppat.1001126 20941354 PMC2947996

[B48] Martínez P. Ljungdahl P. O. (2005). Divergence of Stp1 and Stp2 transcription factors in *Candida albicans* places virulence factors required for proper nutrient acquisition under amino acid control. Mol. Cell. Biol. 25, 9435–9446. 10.1128/MCB.25.21.9435-9446.2005 16227594 PMC1265835

[B49] Meis J. F. Chowdhary A. (2018). Candida auris: a global fungal public health threat. Lancet Infect. Dis. 18, 1298–1299. 10.1016/S1473-3099(18)30609-1 30293876

[B50] Mizusawa M. Miller H. Green R. Lee R. Durante M. Perkins R.. (2017). Can Multidrug-Resistant *Candida auris* Be Reliably Identified in Clinical Microbiology Laboratories? J. Clin. Microbiol. 55, 638–640. 10.1128/JCM.02202-16 27881617 PMC5277535

[B51] Muñoz J. F. Gade L. Chow N. A. Loparev V. N. Juieng P. Berkow E. L.. (2018). Genomic insights into multidrug-resistance, mating and virulence in *Candida auris* and related emerging species. Nat. Commun. 9, 1–13. 10.1038/s41467-018-07779-6 30559369 PMC6297351

[B52] Naglik J. R. Challacombe S. J. Hube B. (2003). *Candida albicans* secreted aspartyl proteinases in virulence and pathogenesis. Microbiol. Mol. Biol. Rev. 67, 400–428. 10.1128/MMBR.67.3.400-428.2003 12966142 PMC193873

[B53] Niimi K. Maki K. Ikeda F. Holmes A. R. Lamping E. Niimi M.. (2006). Overexpression of Candida albicans CDR1, CDR2, or MDR1 Does Not Produce Significant Changes in Echinocandin Susceptibility. Antimicrob. Agents Chemother. 50, 1148–1155. 10.1128/AAC.50.4.1148-1155.2006 16569823 PMC1426986

[B54] Pande K. Chen C. Noble S. M. (2013). Passage through the mammalian gut triggers a phenotypic switch that promotes *Candida albicans* commensalism. Nat. Genet. 45, 1088–1091. 10.1038/ng.2710 23892606 PMC3758371

[B55] Pasrija R. Banerjee D. Prasad R. (2007). Structure and function analysis of CaMdr1p, a major facilitator superfamily antifungal efflux transporter protein of *Candida albicans*: identification of amino acid residues critical for drug/H+ transport. Eukaryot Cell 6, 443–453. 10.1128/EC.00315-06 17209122 PMC1828935

[B56] Prasad R. De Wergifosse P. Goffeau A. Balzi E. (1995). Molecular cloning and characterization of a novel gene of *Candida albicans*, CDR1, conferring multiple resistance to drugs and antifungals. Curr. Genet. 27, 320–329. 10.1007/BF00352101 7614555

[B57] Prasad R. Nair R. Banerjee A. (2019). Multidrug transporters of Candida species in clinical azole resistance. Fungal Genet. Biol. 132, 103252. 10.1016/j.fgb.2019.103252 31302289

[B58] Rakita A. Nikolić N. Mildner M. Matiasek J. Elbe-Bürger A. (2020). Re-epithelialization and immune cell behaviour in an ex vivo human skin model. Sci. Rep. 10, 1. 10.1038/s41598-019-56847-4 31913322 PMC6959339

[B59] Reuß O. Vik Å. Kolter R. Morschhäuser J. (2004). The SAT1 flipper, an optimized tool for gene disruption in *Candida albicans* . Gene 341, 119–127. 10.1016/j.gene.2004.06.021 15474295

[B60] Richards E. J. (2006). Inherited epigenetic variation — revisiting soft inheritance. Nat. Rev. Genet. 7, 395–401. 10.1038/nrg1834 16534512

[B61] Ritz C. Baty F. Streibig J. C. Gerhard D. (2016). Dose-Response Analysis Using R. PloS One 10, e0146021. 10.1371/journal.pone.0146021 PMC4696819 26717316

[B62] Robinson M. D. McCarthy D. J. Smyth G. K. (2009). edgeR: a Bioconductor package for differential expression analysis of digital gene expression data. Bioinformatics 26, 139–140. 10.1093/bioinformatics/btp616 19910308 PMC2796818

[B63] Rybak J. M. Doorley L. A. Nishimoto A. T. Barker K. S. Palmer G. E. Rogers P. D. (2019). Abrogation of Triazole Resistance upon Deletion of CDR1 in a Clinical Isolate of *Candida auris* . Antimicrob. Agents Chemother. 63, e00057–e00019. 10.1128/AAC.00057-19 30718246 PMC6437491

[B64] Rybak J. M. Muñoz J. F. Barker K. S. Parker J. E. Esquivel B. D. Berkow E. L.. (2020). Mutations in TAC1B: a Novel Genetic Determinant of Clinical Fluconazole Resistance in *Candida auris* . MBio 11, e00365–e00320. 10.1128/mBio.00365-20 32398311 PMC7218281

[B65] Sanglard D. Kuchler K. Ischer F. Pagani J. L. Monod M. Bille J. (1995). Mechanisms of resistance to azole antifungal agents in *Candida albicans* isolates from AIDS patients involve specific multidrug transporters. Antimicrob. Agents Chemother. 39, 2378–2386. 10.1128/AAC.39.11.2378 8585712 PMC162951

[B66] Satoh K. Makimura K. Hasumi Y. Nishiyama Y. Uchida K. Yamaguchi H. (2009). *Candida auris* sp. nov., a novel ascomycetous yeast isolated from the external ear canal of an inpatient in a Japanese hospital. Microbiol. Immunol. 53, 41–44. 10.1111/j.1348-0421.2008.00083.x 19161556

[B67] Schelenz S. Hagen F. Rhodes J. L. Abdolrasouli A. Chowdhary A. Hall A.. (2016). First hospital outbreak of the globally emerging *Candida auris* in a European hospital. Antimicrob. Resist. Infect. Control 5, 35. 10.1186/s13756-016-0132-5 27777756 PMC5069812

[B68] Schuetzer-Muehlbauer M. Willinger B. Krapf G. Enzinger S. Presterl E. Kuchler K. (2003). The *Candida albicans* Cdr2p ATP-binding cassette (ABC) transporter confers resistance to caspofungin. Mol. Microbiol. 48, 225–235. 10.1046/j.1365-2958.2003.03430.x 12657057

[B69] Schwarzmüller T. Ma B. Hiller E. Istel F. Tscherner M. Brunke S.. (2014). Systematic Phenotyping of a Large-Scale *Candida glabrata* Deletion Collection Reveals Novel Antifungal Tolerance Genes. PloS Pathog. 10, e1004211. 10.1371/journal.ppat.1004211 24945925 PMC4063973

[B70] Sedlazeck F. J. Rescheneder P. von Haeseler A. (2013). NextGenMap: fast and accurate read mapping in highly polymorphic genomes. Bioinformatics 29, 2790–2791. 10.1093/bioinformatics/btt468 23975764

[B71] Sharma C. Kumar N. Pandey R. Meis J. F. Chowdhary A. (2016). Whole genome sequencing of emerging multidrug resistant *Candida auris* isolates in India demonstrates low genetic variation. New Microbes New Infect. 13, 77–82. 10.1016/j.nmni.2016.07.003 27617098 PMC5006800

[B72] Shivarathri R. Jenull S. Stoiber A. Chauhan M. Mazumdar R. Singh A.. (2020). The Two-Component Response Regulator Ssk1 and the Mitogen-Activated Protein Kinase Hog1 Control Antifungal Drug Resistance and Cell Wall Architecture of *Candida auris* . MSphere 5, e00973–e00920. 10.1128/mSphere.00973-20 33055262 PMC7565899

[B73] Silver P. M. Oliver B. G. White T. C. (2004). Role of *Candida albicans* transcription factor Upc2p in drug resistance and sterol metabolism. Eukaryot Cell 3, 1391–1397. 10.1128/EC.3.6.1391-1397.2004 15590814 PMC539032

[B74] Skelly D. A. Merrihew G. E. Riffle M. Connelly C. F. Kerr E. O. Johansson M.. (2013). Integrative phenomics reveals insight into the structure of phenotypic diversity in budding yeast. Genome Res. 23, 1496–1504. 10.1101/gr.155762.113 23720455 PMC3759725

[B75] Sorgo A. G. Brul S. de Koster C. G. de Koning L. J. Klis F. M. (2013). Iron restriction-induced adaptations in the wall proteome of *Candida albicans* . Microbiology 159, 1673–1682. 10.1099/mic.0.065599-0 23728625

[B76] Szekely A. Borman A. M. Johnson E. M. (2019). *Candida auris* Isolates of the Southern Asian and South African Lineages Exhibit Different Phenotypic and Antifungal Susceptibility Profiles In Vitro. J. Clin. Microbiol. 57, e02055–e02018. 10.1128/JCM.02055-18 30867237 PMC6498034

[B77] Tscherner M. Zwolanek F. Jenull S. Sedlazeck F. J. Petryshyn A. Frohner I. E.. (2015). The *Candida albicans* Histone Acetyltransferase Hat1 Regulates Stress Resistance and Virulence via Distinct Chromatin Assembly Pathways. PloS Pathog. 11, 1–32. 10.1371/journal.ppat.1005218 PMC4608838 26473952

[B78] Wasi M. Khandelwal N. K. Moorhouse A. J. Nair R. Vishwakarma P. Bravo Ruiz G.. (2019). ABC Transporter Genes Show Upregulated Expression in Drug-Resistant Clinical Isolates of Candida auris: A Genome-Wide Characterization of ATP-Binding Cassette (ABC) Transporter Genes. Front. Microbiol. 10, 1445. 10.3389/fmicb.2019.01445 31379756 PMC6647914

[B79] Williams R. B. H. Chan E. K. F. Cowley M. J. Little P. F. R. (2007). The influence of genetic variation on gene expression. Genome Res. 17, 1707–1716. 10.1101/gr.6981507 18063559

[B80] Wu F. Zhao S. Yu B. Chen Y.-M. Wang W. Song Z.-G.. (2020). A new coronavirus associated with human respiratory disease in China. Nature 579, 265–269. 10.1038/s41586-020-2008-3 32015508 PMC7094943

[B81] Yang H. Tong J. Lee C. W. Ha S. Eom S. H. Im Y. J. (2015). Structural mechanism of ergosterol regulation by fungal sterol transcription factor Upc2. Nat. Commun. 6, 6129. 10.1038/ncomms7129 25655993

[B82] Yu G. Wang L.-G. Han Y. He Q.-Y. (2012). clusterProfiler: an R Package for Comparing Biological Themes Among Gene Clusters. Omi A J. Integr. Biol. 16, 284–287. 10.1089/omi.2011.0118 PMC3339379 22455463

[B83] Yue H. Bing J. Zheng Q. Zhang Y. Hu T. Du H.. (2018). Filamentation in *Candida auris*, an emerging fungal pathogen of humans: passage through the mammalian body induces a heritable phenotypic switch. Emerg. Microbes Infect. 7, 1–13. 10.1038/s41426-018-0187-x 30482894 PMC6258701

[B84] Zamith-Miranda D. Heyman H. M. Cleare L. G. Couvillion S. P. Clair G. C. Bredeweg E. L.. (2019). Multi-omics Signature of *Candida auris*, an Emerging and Multidrug-Resistant Pathogen. MSystems 4, e00257–e00219. 10.1128/mSystems.00257-19 31186339 PMC6561322

