# Supplementary material for: Transcriptome Signatures Predict Phenotypic Variations of Candida auris
Source: Front Cell Infect Microbiol. 2021 Apr 14;11:662563. doi: 10.3389/fcimb.2021.662563 (PMC8079977; doi:10.3389/fcimb.2021.662563)
Supplement: Supplementary file 1 [file DataSheet_1.pdf]

Supplementary information

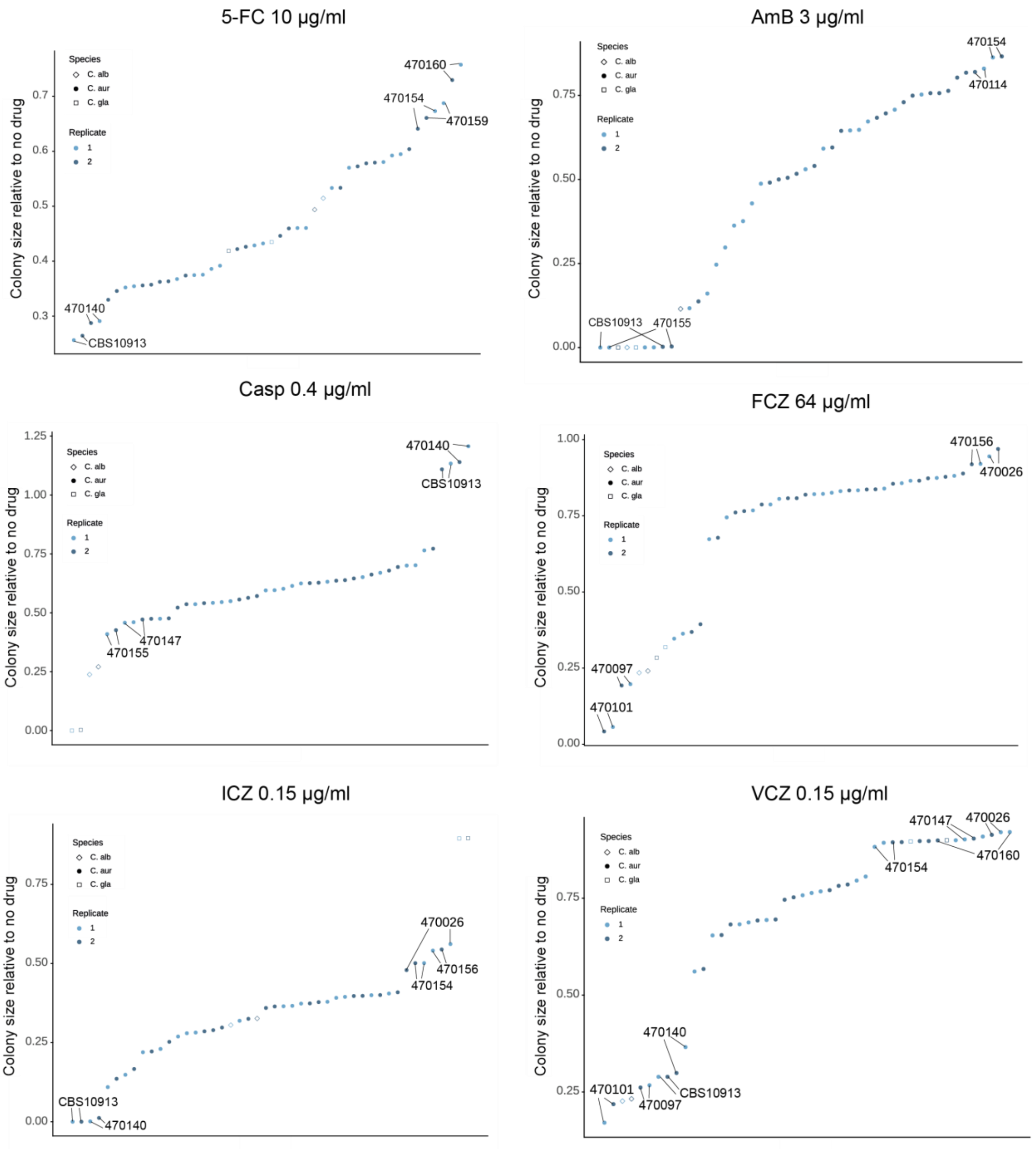

Supplementary Figure 1. Intra-clade variability in antifungal susceptibilities. Quantification of plate-based antifungal susceptibility screening shown in Figure 1A. Colony size in response to the indicated antifungal relative to the colony growth without antifungal was used as a readout for antifungal susceptibility. *C. albicans* SC5314, *C. glabrata* ATCC2001 and clade II *C. auris* CBS10913 were used as additional controls. Colony quantifications from both biological replicates are shown; *C. auris* isolates with low or high antifungal susceptibility are highlighted. 5-FC = 5-fluorcytosine, AmB = amphotericin B, Casp = caspofungin, FCZ = fluconazole, ICZ = itraconazole, VCZ = voriconazole. C. alb = *C.albicans*, C. gla = *C. glabrata*, *C. aur* = *C. auris*. *Related to Figure 1*.

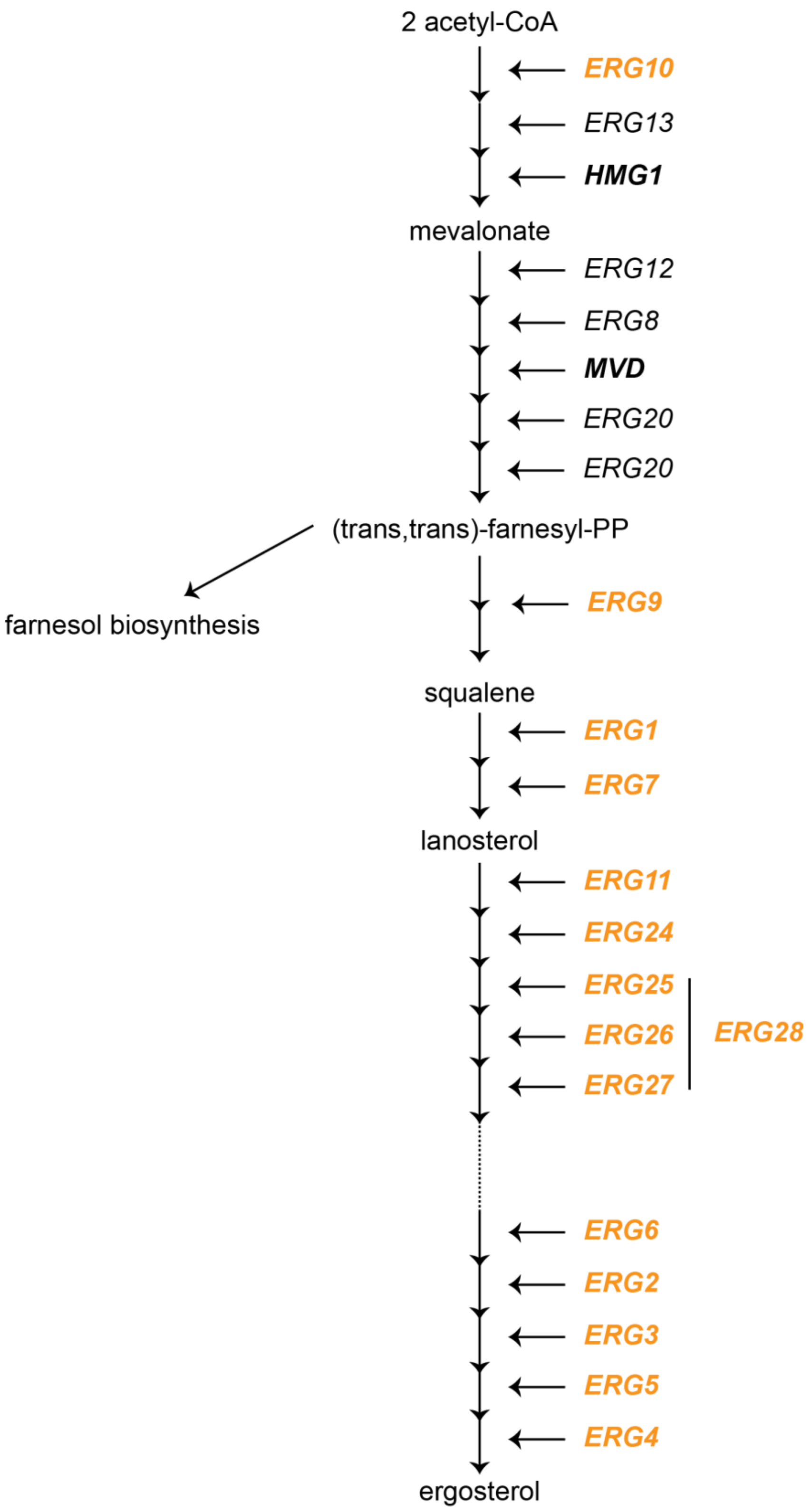

Supplementary Figure 2. Ergosterol biosynthesis pathway. Overview of the ergosterol biosynthesis pathway based on the KEGG database (annotation *C. albicans*, cal00100 and cal00900). Genes upregulated (> 1.5-fold and FDR < 0.05) in all clade I isolates with respect to CBS10913 are depicted in orange. *C. auris* homologues of *C. albicans HMG1* and *MVD* (depicted in bold font) were > 1.5-fold upregulated in isolate 470147 vs CBS10913 and 1.2-1.5-fold upregulated (FDR < 0.05) in 470154/470140 vs CBS10913. *Related to Figure 2*.

Supplementary Tables

Supplementary Table 1: Strains and plasmids used in this study.

Supplementary Table 2: Oligos used in this study

Supplementary Table 3. RNA-seq edgeR analysis result – ***Related to Figure 2***

Supplementary Table 4. Commonly differentially expressed genes (DEGs) and GO enrichment analysis - ***Related to Figure 2***

Supplementary Table 5. Pairwise comparison of 470154 vs 470140 (RNA-seq) – ***Related to Figure 3***

Supplementary Table 6. Upregulated genes in 470154 vs 470140 and GO enrichment analysis result– ***Related to Figure 3***
